# Changes of Electrocardiogram and Myocardial Enzymes in Patients with Intracerebral Hemorrhage

**DOI:** 10.1155/2022/9309444

**Published:** 2022-02-01

**Authors:** Guannan Qin, Chuanyang Dai, Shuang Feng, Guofeng Wu

**Affiliations:** ^1^Guizhou Medical University, Guian New Area University Town, Guizhou Province 550025, China; ^2^Emergency Department, Guizhou Province, The Affiliated Hospital of Guizhou Medical University, No. 28, Guiyijie Road, Liuguangmen, Guiyang City 550004, China; ^3^Emergency Department, Shandong Province, Zibo Central Hospital, No. 54, Gongqingtuan West Road, Zhangdian District, Zibo City 255020, China

## Abstract

**Purpose:**

Cardiac complications are common in patients with spontaneous intracerebral hemorrhage (ICH). The present study is aimed at observing the incidence of cardiac complications after ICH, so as at improving the understanding of the relationship between cardiac complications and ICH.

**Methods:**

This is a retrospective study on analyzing electrocardiogram (ECG) and serum myocardial enzyme of 208 patients with ICH admitted to a tertiary hospital from 2018 to 2019. For each patient, demographics, medical history, clinical presentation, ECG, serum myocardial enzyme, and head CT on admission were reviewed. Mortality was noted.

**Results:**

Among the 208 patients, 145 (69.71%) had one or more ECG abnormalities. The top three abnormalities were corrected QT interval (QTc) prolongation 52 (25%), ST depression 48 (23.08%), and T wave inversion 38 (18.27%). One hundred and thirty-nine patients (66.83%) had increased serum levels of at least one kind of myocardial enzyme, which were high-sensitive cardiac troponin T (hs-cTnT) 79 (37.98%), lactic dehydrogenase (LDH) 80 (38.46%), creatine kinase (CK) 57 (27.40%), and creatine kinase-myocardial subfraction (CKMB) 57 (27.40%). The logistic regression analysis showed the following: secondary intraventricular hemorrhage (SIVH) (odds ratio (OR) 5.32; 95% confidence interval (CI) 2.55–11.08; *p* < 0.001) and hematoma volume > 30 ml (OR 3.81; 95% CI 1.86–7.81; *p* < 0.001) were independent predictive factors of QTc prolongation; thalamus location (OR 5.79; 95% CI 1.94–17.28; *p* < 0.05), hematoma volume > 30 ml (OR 24.187; 95% CI 3.14-186.33; *p* < 0.05), insular involvement (OR 19.08; 95% CI 5.77-63.07; *p* < 0.001), and SIVH (OR 2.62; 95% CI 1.69-5.86; *p* < 0.05) were independent predictive factors of ST depression; insular involvement (OR 2.90; 95% CI 1.12–7.50; *p* < 0.05) and hematoma volume > 30 ml (OR 1.98; 95% CI 1.06–3.70; *p* < 0.05) were independent predictive factors of increase of CK; Glasgow Coma Scale (GCS) (OR 0.86; 95% CI 0.78–0.98; *p* < 0.05) and insular involvement (OR 5.56; 95% CI 1.98–15.62; *p* < 0.05) were independent predictive factors of increase of CKMB; SIVH (OR 2.05; 95% CI 1.07–3.92; *p* < 0.05) was independent predictive factor of increase of LDH; age (OR 1.03; 95% CI 1.01–1.06; *p* < 0.05), blood glucose on admission (OR 1.10; 95% CI 1.01–1.20; *p* < 0.05), and history of antiplatelet drug use (OR 3.50; 95% CI 1.01–12.12; *p* < 0.05) were independent predictive factors of hs-cTnT. All the injury indexes were not related to in-hospital mortality.

**Conclusion:**

The study suggests that insular involvement, hematoma volume > 30 ml, and SIVH are the strongest risk factors for ECG abnormalities and elevated myocardial enzymes after ICH followed which are the risk factors such as GCS, age, admission blood glucose, and ICH location in the thalamus.

## 1. Introduction

Despite advances in stroke care management, spontaneous intracerebral hemorrhage (ICH) is still a kind of serious stroke with high morbidity and mortality [1]. Secondary systemic complications are common and largely affect the outcomes of patients with ICH [2]. It is extremely important to identify and manage complications associated with clinical outcomes after ICH.

In fact, cardiac complications are frequently found in patients with acute stroke. Relevant studies mainly focused on ischemic stroke and subarachnoid hemorrhage, but few studies are on ICH [3, 4]. A study has shown that cardiac complications may occur in 1% to 4% of patients with ICH [5]. Markers of myocardial injury in the complications, such as myocardial markers and electrocardiogram (ECG), are associated with prognosis in patients with ICH [6, 7]. It is reported that ECG abnormalities after ICH are very common (56%-81%) [8, 9], which includes prolonged corrected QT (QTc) interval, ST-T changes, arrhythmias, and atrioventricular nodal blocks [10]. The most common cardiac biomarker in patients with ICH is troponin I [11]. The 2015 American Heart Association/American Stroke Association ICH guidelines recommend cardiac workup of patients with spontaneous ICH, including both ECG and cardiac troponin, to assess active coronary ischemia and concomitant myocardial injury [12].

However, little is known about the relationship between these markers and various characteristics of ICH [13]. Therefore, we investigated the prevalence of myocardial injury in patients with ICH and the possible associations with clinical and radiological findings. Our study aimed at improving the understanding of the possible association between myocardial injury and clinical characteristics in patients with ICH. Furthermore, it could be also helpful to identify high-risk patients and predict possible adverse events.

## 2. Materials and Methods

### 2.1. Patients

**Figure 1:**
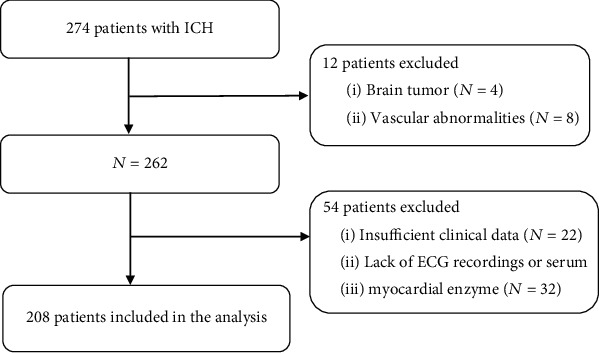
Research flow chart.

Ethics approval was obtained from the local institutional review board (NO. S2017-02). Because it was a retrospective study, no written informed consent was required. Between 2018 and 2019, 274 patients with spontaneous ICH were admitted to the Emergency Neurology Department, Guizhou Medical University. General inclusion criteria contained spontaneous ICH confirmed by computed tomography (CT) scan, with the age being older than 18 years. Exclusion criteria were CT angiography (CTA) or magnetic resonance angiography (MRA) diagnosis of ICH due to brain tumor or vascular abnormalities, insufficient clinical data, ECG recordings, and serum myocardial enzyme on admission. Sixty-six patients were excluded due to brain tumor (*N* = 4), vascular abnormalities (*N* = 8), insufficient clinical data (*N* = 22), and lack of ECG recordings or serum myocardial enzyme (*N* = 32). Therefore, the subjects of the study included 208 patients in total (Figure 1).

### 2.2. Data Collection

The data were retrospectively analyzed to determine such clinical characteristics of the patients as age, sex, medical history, time of onset at admission, Glasgow Coma Scale (GCS), location of hematoma, side of hematoma, presence of insular involvement, secondary intraventricular hemorrhage (SIVH), hematoma volume, blood tests (hemoglobin, platelet, white cell, and coagulation level), ECG findings, and level of serum myocardial enzyme. The location of hematoma was classified into basal ganglia, lobar, thalamus, cerebellum, brain stem, and primary intraventricular hemorrhage (PIVH). Hematoma volume was measured through the ABC/2 method. Within 1 hour after admission, laboratory tests (blood tests, ECG, etc.) were performed for all involved patients. Mortality during hospitalization was reviewed.

### 2.3. ECG Analyses

ECG (12-lead) recordings were collected on admission before treatment at a paper speed of 25 millimeters per second, with an amplitude calibration of 10 millimeters per millivolt, and were analyzed by the same attending cardiologist, who did not know the details of the patients. Abnormalities of ECG are judged by the following criteria [6]: (a) rhythm: sinus rhythm, atrial fibrillation, or ectopic beats; (b) heart rate: sinus bradycardia (<60 beats/minute) and sinus tachycardia (>100 beats/min); (c) PR interval: short <0.12 seconds and prolonged >0.2 seconds; (d) QRS complex width: prolonged >0.12 seconds; (e) corrected QT interval (QTc) (using the Bazett formula): prolonged at >0.45 seconds in females and >0.44 seconds in males; (f) ST segment depression: downsloping or horizontal > 0.05 millivolts; (g) ST segment elevation: convexity of the ST segment upward > 0.1 millivolts; and (h) T wave inversion: negative T wave of ≥1 millimeters in depth in two or more contiguous leads, with exclusion of leads aVR, III, and V1.

### 2.4. The Analysis of Serum Myocardial Enzyme

The elevation standard of myocardial enzyme is judged according to the reference range of our hospital's laboratory: (a) high creatine kinase (CK) value: >198 U/l; (b) high creatine kinase-myocardial subfraction (CKMB) value: >25 U/l; (c) high lactic dehydrogenase (LDH) value: >250 U/l; and (d) high-sensitive cardiac troponin T (hs-cTnT) value: >0.014 ng/ml.

### 2.5. Other Blood Tests

The standards for these tests also followed the hospital's laboratory criteria: (a) leukocytosis: white blood cell count > 10 × 10^9^/l; (b) low platelet count: platelet count < 100 × 10^9^/l; (c) abnormal hemoglobin level: hemoglobin levels up or down; and (d) coagulation abnormalities: one or more of the terms of prothrombin time (PT), activated partial thromboplastin time (APTT), international normalized ratio (INR), and D-dimer and fibrinogen up or down.

### 2.6. Statistical Analysis

The descriptive statistics are expressed as the mean ± standard deviation (SD) or medians with interquartile ranges (IQR) for the continuous variables, while the categorical variables are expressed as percentages, as appropriate. Univariate analysis was used to determine the possible relationships between each ECG abnormality and clinical features, as well as each elevated serum myocardial enzyme and clinical features, by means of either the chi-square test or Fisher's exact test. If an ECG abnormality was observed in more than 10% of patients, it was analyzed.

The relationship between in-hospital mortality and markers of cardiac injury, including ECG abnormalities observed in more than 10% of patients and serum myocardial enzymes, was also analyzed using the methods mentioned above.

The analysis of multivariate logistic regression was used to determine the independent correlation factors with ECG abnormalities and elevated serum myocardial enzymes and mortality. The variables with a significant level of *p* < 0.05 in the univariate analysis were included as independent variables in the logistic regression analyses. Only those variables with *p* < 0.05 in two-tailed tests were retained within the model. Odds ratios (OR) and 95% confidence intervals (CI) were reported. A value of *p* < 0.05 was regarded as statistical significance. All statistical analysis was conducted by virtue of SPSS (version 22.0; IBM Corporation, Armonk, NY, USA).

## 3. Results

The results of the clinical information, level of myocardial enzyme, and imaging findings of 208 patients with ICH are summarized in Table 1. There were 136 males (65.38%) and 72 females, with a mean age of 61.23 years (range: 29-93). The GCS was ≤8 in 60 cases (28.85%). Past history included hypertension in 140 cases (67.31%), type 2 diabetes in 16 (7.69%), cerebrovascular diseases in 32 (15.38%), coronary heart disease in 11 (5.29%), smoking history in 76 (36.54%), drinking history in 45 (21.63%), history of antiplatelet drug use in 14 (6.73%), and anticoagulant drug use in 1 (0.48%). The mean time of onset on admission was 6 hours (3, 24). The mean blood glucose was 7.43 mmol/l (5.93, 9.97) on admission. Hematoma was located in the basal ganglia in 100 cases (48.08%), lobar region in 62 (29.81%), thalamus in 28 (13.46%), cerebellum in 11 (5.29%), brain stem in 4 (1.92%), and primary intraventricular hemorrhage in 1 (0.48%). Hematoma involved the insular lobe in 20 cases (9.62%) and secondary intraventricular hemorrhage (26.92%) in 56 cases. 96 cases (46.15%) had hematoma on the right side. The hematoma was >30 ml in 87 cases (41.83%), and the mean volume was 27 ml (10, 50). The blood routine tests showed leukocytosis in 8 cases (3.85%), low platelet count in 2 cases (0.96%), abnormal hemoglobin level in 3 cases (1.44%), and coagulation abnormalities in 2 cases (0.96%). One or more ECG abnormalities were observed in 145 cases (69.71%). The most common ECG abnormality was QTc prolongation (25.00%), followed by ST segment depression (23.08%) and T wave inversion (18.27%). Elevated levels of one more kind of abnormal myocardial enzymes were observed in 139 patients (66.83%). The most common type was LDH (38.4%), followed by hs-cTnT (37.9%), CK 57 (27.40%), and CKMB 57 (27.40%). Relevant ECG abnormalities that reached at least 10% of prevalence in the cohort were included in a univariate analysis and are illustrated in Tables 2 and 3. Relevant elevated myocardial enzymes were included in a univariate analysis and are illustrated in Tables 2 and 4.

### 3.1. Analysis of QTc Prolongation

The univariate analysis presented that QTc prolongation was associated with location of the cerebellum (*p* = 0.049), SIVH (*p* < 0.001), and hematoma volume > 30 ml (*p* < 0.001). The logistic regression analysis showed that SIVH (OR 5.32; 95% CI 2.55–11.08; *p* < 0.001) and hematoma volume > 30 ml (OR 3.81; 95% CI 1.86–7.81; *p* < 0.001) were independent predictive factors of QTc prolongation.

### 3.2. Analysis of ST Depression

The univariate analysis showed that ST depression was associated with GCS ≤ 8 (*p* = 0.009), location of the thalamus (*p* = 0.029), insular involvement (*p* < 0.001), SIVH (*p* = 0.009), and hematoma volume > 30 ml (*p* = 0.008). The logistic regression analysis depicts that location of the thalamus (OR 5.79; 95% CI 1.94–17.28; *p* < 0.05), hematoma volume > 30 ml (OR 3.68; 95% CI 1.40–9.67; *p* < 0.05), insular involvement (OR 19.08; 95% CI 5.77–63.07; *p* < 0.001), and SIVH (OR 2.62; 95% CI 1.17–5.86; *p* < 0.05) were independent predictive factors of ST depression.

### 3.3. Analysis of Inverted T Wave

The univariate analysis showed that inverted T wave was associated with age (*p* = 0.006), history of cerebrovascular diseases (*p* = 0.01), history of antiplatelet drug use (*p* = 0.001), location of basal ganglia (*p* < 0.001), lobar (*p* < 0.001), and thalamus (*p* < 0.001). The logistic regression analysis showed that there was no independent predictive factor of inverted T wave.

### 3.4. Analysis of LBBB

The univariate analysis showed that LBBB was associated with GCS (*p* = 0.003), primary intraventricular hemorrhage (*p* = 0.046), hematoma volume > 30 ml (*p* = 0.001), and abnormal hemoglobin level (*p* < 0.001). The logistic regression analysis showed that there was no independent predictive factor of LBBB.

### 3.5. Analysis of Increase in CK

The univariate analysis showed that the increase in CK was associated with insular involvement (*p* = 0.017) and hematoma volume > 30 ml (*p* = 0.024). The logistic regression analysis showed that insular involvement (OR 2.9; 95% CI 1.12–7.50; *p* < 0.05) and hematoma volume > 30 ml (OR 1.98; 95% CI 1.06–3.70; *p* < 0.05) were independent predictive factors of the increase in CK.

### 3.6. Analysis of Increase in CKMB

The univariate analysis showed that the increase in CKMB was associated with time of onset at admission (*p* = 0.039), GCS (*p* < 0.001), blood glucose (*p* = 0.02), and insular involvement (*p* < 0.001). The logistic regression analysis showed that GCS (OR 0.87; 95% CI 0.79–0.95; *p* < 0.05) and insular involvement (OR 5.57; 95% CI 1.98–15.64; *p* = 0.001) were independent predictive factors of the increase in CKMB.

### 3.7. Analysis of Increase in LDH

The univariate analysis showed that the increase in LDH was associated with GCS (*p* = 0.013), blood glucose (*p* = 0.008), SIVH (*p* = 0.007), and hematoma volume > 30 ml (*p* = 0.006). The logistic regression analysis showed that SIVH (OR 2.05; 95% CI 1.071–3.92; *p* < 0.05) was an independent predictive factor of the increase in LDH.

### 3.8. Analysis of Increase in hs-cTnT

The univariate analysis showed that the increase in hs-cTnT was associated with age (*p* = 0.003), history of T2D (type 2 diabetes) (*p* = 0.008), history of antiplatelet drug use (*p* = 0.007), blood glucose (*p* = 0.024), location of basal ganglia (*p* = 0.046), and leukocytosis (*p* = 0.025). The analysis of logistic regression showed that age (OR 1.03; 95% CI 1.01-1.06; *p* < 0.05), blood glucose (OR 1.10; 95% CI 1.01-1.20; *p* < 0.05), and history of antiplatelet drug use (OR 3.504; 95% CI 1.01-12.12; *p* < 0.05) were independent predictive factors of the increase in hs-cTnT.

### 3.9. Impact of Cardiac Injury Markers on Mortality

In a univariate analysis, there was no ECG abnormality observed in more than 10% of patients and elevated serum myocardial enzyme associated with in-hospital mortality.

## 4. Discussion

Many researchers have observed the changes of ECG and myocardial enzymes in ICH patients. However, their studies only attach the importance to one or two markers of ECG and myocardial enzymes, the analysis on both of which is less researched in the same group of patients. Therefore, it is impossible to fully discover what factors affect the heart after ICH. In this study, ECG and myocardial enzymes of 208 patients with ICH were analyzed together so as to further clarify the relationship between different characteristics of ICH and cardiac complications. The result showed that insular involvement, hematoma volume > 30 ml, and SIVH were the strongest risk factors triggering the abnormalities of ECG and myocardial enzyme after ICH, followed by GCS, age, admission blood glucose, and location of the thalamus.

ECG abnormalities (69.71%) were common in patients with ICH in our study, which was basically consistent with the incidence reported in literature [8, 9]. The most common ECG abnormalities were prolonged QTC, ST segment depression, and inverted T wave. Some researchers have also found that these abnormalities were often observed during the acute phase of ICH [14]. Then, we examined the factors associated with ECG abnormalities. We found that hematoma volume and SIVH were the two strongest determinants of ECG abnormalities, which is consistent with the previous reports [15].

In the study, elevated myocardial enzymes (74.52%) were also found in the plasma of patients with ICH, among which, LDH was the most common one, followed by hs-cTnT. Studies have shown that LDH levels are increased in patients with central nervous system diseases such as cerebral infarction and hypoxic-ischemic encephalopathy [16, 17], as well as ICH [18]. The proportion of increased troponin in this study (37.98%) is higher than that in other studies (27.4%) [6] and (22%) [19]. It seems to have to do with the inconsistent types of cardiac troponin in the two studies. hs-cTnT was studied in this study while cTnI or cTnT in others. We examined the risk factors associated with the four myocardial enzymes observed. Insular involvement was the main determinant of myocardial enzyme elevation, related to two kinds. In addition, GCS, hematoma volume, SIVH, age, and blood glucose levels were also associated with one item, respectively. This is consistent with many studies, in which myocardial enzyme correlated with markers of disease severity such as hematoma volume, NIHSS score, and intraventricular hemorrhage [20–22].

Insula is important to cardiac sympathetic output maintenance. ICH patients whose insula was involved had an increased risk of cardiac complications [23], which is consistent with our findings. It also showed that the risk was elevated when the right hemisphere was involved. Although there is evidence that insular cortex-mediated sympathetic control is hemispheric dominant [24], the experimental results are still inconsistent [25]. However, our results did not show a difference in ECG or serum myocardial enzyme effects between left and right hemisphere hematoma. The reason may be that our study was a clinical retrospective study, and the results were derived from animal experiments. Therefore, the hematoma location identified in our study is not as accurate as in animal studies, which seems to influence judgments of hemispheric laterality.

Hematoma extension to the third and fourth ventricles may trigger destruction of the baroreflex receptors around the area [26], because of the abundant network structures controlling autonomic nerve function around the third and fourth ventricles, involving the paraventricular nucleus of the hypothalamus, periaqueductal gray matter, brain stem, and so on. Expansion of the intraventricular hematoma may lead to acute autonomic nervous system disorders and elevated circulating catecholamine levels, which could lead to cardiac complications [27]. In addition, the central control of the autonomic nervous system was thought to be located in the insular cortex, cingulate gyrus, amygdala, and hypothalamus [28]. Cardiovascular autonomic centers may also involve the extrainsular regions and their interconnecting fibers [29]. Therefore, the larger the hematoma, the more severe the pressure on the central control area, and the more severe the damage to the contact fibers. This also increases the risk of heart complications and explains why both hematoma volume > 30 ml and SIVH were independent risk factors for ECG and myocardial enzyme abnormities.

Our study found that lower GCS was closely related to the increase of CKMB after ICH and also an independent predictor. However, in patients with traumatic brain injury (TBI), researchers did not find association between GCS and the development of cardiac dysfunction [29]. An explanation for the difference could be that the damage of hematoma in ICH to the brain tissue is limited, while it is diffused in TBI. This kind of diffuse lesion may mask the effect of the autonomic nerve center on the heart. The study confirmed that cardiac complications were more frequent with increasing grading of diffuse brain injury [30]. In addition, GCS reflects the functional integrity of the brain stem. And the brain stem, especially the caudal autonomic centers, is very important for the nervous influences to the heart. One study observed the heart rate variability (HRV) in patients in coma due to different diseases. HRV was found a progressive trend associated with deepening of coma, assessed by the GCS [31]. This is consistent with our findings, asserting that GCS could predict the occurrence of cardiac complications in central nervous system diseases.

We demonstrated that location of the thalamus is an independent risk factor for ST depression. This is related to the fact that the thalamus is a relay station for autonomous afferent impulses to the insular cortex [32] and is also involved in the autonomic nervous system pathway [33]. Racho et al. had also noted the importance of the thalamus in the regulation of the ANS, through the observation of Takotsubo cardiomyopathy after a thalamic stroke [34].

Elevated troponin is frequently detected in patients with stroke [6, 35]. In this study, admission blood glucose level, age, and history of antiplatelet drug use were found to be risk factors for elevated troponin. Type 2 diabetes has been proven to have a significant association with elevated hs-cTnT levels in community-dwelling population; fasting blood glucose played a crucial role in this association [36]. Sharain et al. studied 830 patients and found that older age was also associated with elevated hs-cTnT [37]. We believe that admission blood glucose and age also affect the increase of troponin after ICH. The main reason why blood glucose and age affect the increase of troponin after ICH may be that hyperglycemia and old age reduce the cardiac tolerance of patients and are also prone to cardiac complications such as myocardial ischemia or necrosis after ICH.

History of antiplatelet drug use means those patients suffered from severe atherosclerosis, such as coronary artery occlusion. Scheitz et al. [38] believed that troponin may be chronically elevated or acutely elevated in patients with stroke, which needs to be determined by further evaluation. Thus, in our study, troponin may be chronically elevated in the patients with a history of antiplatelet drugs. The cause may be the coronary factors.

The relationship between injury markers and in-hospital mortality was further investigated. Compared to the published literature, alteration of the injury markers in patients with ICH was not found to be associated with in-hospital mortality. The previous reports showed an association between ECG abnormalities and outcome. However, it is arguable which abnormality can predict the prognosis. Some studies have shown that QT interval could predict the mortality of ICH patients [7, 39]. But other researchers disagreed with this hypothesis. In their study, inversion of T waves was the only ECG aberrancy that is related to the clinical outcome [9]. The same situation also occurs in the relationship between myocardial enzymes and clinical prognosis. Some argued that elevation in cardiac troponin levels has been associated with in-hospital mortality [11, 40, 41], but other studies suggested the opposite case [42]. The possible reason for the inconsistency of the results is that multiple factors independent from ICH could affect ECG and myocardial enzymes, such as medicine, medical history, and structural cardiac pathologies. As these variables could not be controlled completely in all the current researches, any conclusions drawn from this topic are not fully convincing and precise. Our research is no exception. Therefore, it is still uncertain what injury markers could accurately predict the prognosis until a better research method is designed. Another reason of our failure in discovering the correlation between cardiac injury markers and prognosis may be the choice of endpoint observed in this study. The purpose of this study was to determine factors for ECG and increased myocardial enzyme after ICH, so we only analyzed in-hospital mortality. If we were able to include outcomes at 3-6 months, we might have a different finding.

This study had important clinical implications. The ECG and myocardial enzymes of the same group of ICH patients at admission were observed. The possible independent predictors of the early cardiac injury markers were analyzed, albeit this study did not find the relationship between these markers and in-hospital mortality. Our findings are still conducive for clinicians to early identify those patients with ICH who are at high risk of secondary heart injury and intervene in advance. This may improve the prognosis of the patients, but further research is needed.

### 4.1. Limitations

The study is limited in respect of retrospective feature. The single-center study reflected the cardiac complications associated with ICH in the specific patients of Guiyang. Therefore, the results may not be promoted in different groups of patients. It also had a small sample size, which reduced the statistical power and increased the chance of type 2 errors. Additionally, the dynamic changes of ECG and cardiac enzymes were not included as parameter into our study, resulting in a lack of data on whether these changes were transient or permanent. Due to the lack of complete outcome measures for the patients such as mortality at 3 to 6 months and scores from the Glasgow Outcome Scale or Modified Rankin Scale, this resulted in possibly ambiguous associations between cardiac injury markers and prognosis. Despite the limitations, our findings allow for some useful clinical speculations.

## 5. Conclusion

We studied ECG abnormalities and changes of serum myocardial enzymes and the relationship between these markers of cardiac damage and the detailed characteristics of ICH in a series of patients and demonstrated that insular involvement, hematoma volume > 30 ml, and SIVH were the strongest risk factors for ECG and myocardial enzyme abnormalities after ICH. The risk factors are as follows: GCS, age, admission blood glucose, and location of the thalamus. Further prospective studies are needed to corroborate our findings.

## Figures and Tables

**Table 1:** Baseline characteristics of 208 patients with ICH.

Characteristics	Patient data
Age	
Years (SD)	61.23 (13.64)
≤60 years, *n* (%)	110 (52.88)
Male, *n* (%)	136 (65.38)
History, *n* (%)	
History of hypertension	140 (67.31)
History of type 2 diabetes	16 (7.69)
History of cerebrovascular diseases	32 (15.38)
History of coronary heart disease	11 (5.29)
History of smoking	76 (36.54)
History of drinking	45 (21.63)
Antiplatelet drugs	14 (6.73)
Anticoagulant drugs	1 (0.48)
Time, h (IQR)	6 (21)
GCS ≤ 8, *n* (%)	60 (28.85)
Blood glucose, mmol/l (IQR)	7.43 (4.04)
Location of hematoma, *n* (%)	
Basal ganglia	100 (48.08)
Lobar	62 (29.81)
Thalamus	28 (13.46)
Cerebellum	11 (5.29)
Brain stem	4 (1.92)
Primary intraventricular hemorrhage	1 (0.48)
Right-sided hematoma, *n* (%)	96 (46.15)
Insular involvement, *n* (%)	20 (9.62)
Secondary intraventricular hemorrhage, *n* (%)	56 (26.92)
Hematoma volume	
Median, ml (IQR)	27 (40)
>30 ml, *n* (%)	87 (41.83)
Other blood tests, *n* (%)	
Leukocytosis	8 (3.85)
Low platelet count	2 (0.96)
Abnormal hemoglobin level	3 (1.44)
Coagulation abnormalities	2 (0.96)
ECG abnormalities, *n* (%)	
QTc prolongation	52 (25.00)
ST depression	48 (23.08)
Inverted T wave	38 (18.27)
LBBB	11 (5.29)
PVC	8 (3.85)
RBBB	3 (1.44)
Atrial fibrillation	2 (0.96)
Myocardial enzymes values (IQR)	
CK, U/l	122.25 (129.05)
CKMB, U/l	19.4 (12.14)
LDH, U/l	233 (84.5)
hs-cTnT, ng/ml	0.012 (0.011)
Elevated myocardial enzymes, *n* (%)	
CK	57 (27.4)
CKMB	57 (27.4)
LDH	80 (38.46)
hs-cTnT	79 (37.98)
Mortality, *n* (%)	17 (8.17)

ICH: intracerebral hemorrhage; SD: standard deviation; Time: time of onset on admission; GCS: Glasgow Coma Scale; ECG: electrocardiogram; QTc: corrected QT interval; LBBB: left bundle branch block; PVC: premature ventricular contraction; RBBB: right bundle branch block; CK: creatine kinase; CKMB: creatine kinase-myocardial subfraction; LDH: lactic dehydrogenase; hs-cTnT: high-sensitive cardiac troponin T.

**Table 2:** Univariate analysis of significant ECG changes and elevated myocardial enzymes in ICH. Significant values (*p* < 0.05) are in bold.

Variable	QTc prolongation (*n* = 52)	ST depression (*n* = 48)	Inverted T wave (*n* = 38)	LBBB (*n* = 11)	Elevated CK (*n* = 57)	Elevated CKMB (*n* = 57)	Elevated LDH (*n* = 80)	Elevated hs-cTnT (*n* = 79)
Age (SD)	60.37 (13.49)	61.79 (12.38)	**66.92 (13.50)** ^∗^	60.18 (11.80)	60.58 (13.63)	58.42 (13.57)	59.66 (14.20)	**64.91 (14.81)** ^∗^
Male (%)	34 (65.38)	33 (68.75)	20 (52.63)	8 (72.73)	42 (73.68)	43 (75.44)	49 (61.25)	51 (64.56)
History of hypertension (%)	37 (71.15)	33 (68.75)	28 (73.68)	9 (81.82)	39 (68.42)	39 (68.42)	54 (67.50)	57 (72.15)
History of type 2 diabetes (%)	6 (11.54)	4 (8.33)	2 (5.26)	1 (9.09)	5 (8.77)	4 (7.02)	4 (5.00)	**11 (13.92)** ^∗^
History of cerebrovascular diseases (%)	9 (17.31)	10 (20.83)	**11 (28.95)** ^∗^	1 (9.09)	12 (21.05)	10 (17.54)	10 (12.50)	14 (17.72)
History of coronary heart disease (%)	2 (3.85)	2 (4.17)	4 (10.53)	2 (18.18)	3 (5.26)	4 (7.02)	5 (6.25)	7 (8.86)
History of smoking (%)	19 (36.54)	19 (39.58)	11 (28.95)	3 (27.27)	22 (38.60)	25 (43.86)	28 (35.00)	28 (35.44)
History of drinking (%)	11 (21.15)	11 (22.92)	4 (10.53)	2 (18.18)	10 (17.54)	13 (22.81)	15 (18.75)	16 (20.25)
History of antiplatelet drugs (%)	5 (9.62)	5 (10.42)	**7 (18.42)** ^∗^	0 (0.00)	7 (12.28)	7 (12.28)	7 (8.75)	**10 (12.82)** ^∗^
History of anticoagulant drugs (%)	0 (0.00)	1 (2.08)	1 (2.63)	0 (0.00)	0 (0.00)	0 (0.00)	0 (0.00)	0 (0.00)
Time (IQR)	5.00 (18.25)	6.50 (17.50)	6 (21)	5 (5)	7 (21)	**5 (6)** ^∗^	5 (11)	6 (21)
GCS ≤ 8 (%)	32 (61.54)	**27 (56.25)** ^∗^	24 (63.16)	**3 (27.27)** ^∗^	37 (64.91)	**30 (52.63)** ^∗^	**49 (61.25)** ^∗^	51 (64.56)
Blood glucose (IQR)	7.93 (4.01)	7.94 (5.43)	7.065 (3.60)	10.39 (5.34)	7.91 (3.43)	**8.03 (4.09)** ^∗^	**7.97 (4.55)** ^∗^	**7.91 (4.61)** ^∗^
Basal ganglia (%)	26 (50.00)	21 (43.75)	**8 (21.05)** ^∗^	8 (72.73)	27 (47.37)	26 (45.61)	42 (52.50)	**31 (39.24)** ^∗^
Lobar (%)	18 (34.62)	16 (33.33)	**2 (5.26)** ^∗^	1 (9.09)	20 (35.09)	18 (31.58)	23 (28.75)	28 (35.44)
Thalamus (%)	7 (13.46)	**11 (22.92)** ^∗^	**28 (73.68)** ^∗^	0 (0.00)	6 (10.53)	10 (17.54)	11 (13.75)	14 (17.72)
Cerebellum (%)	**0 (0.00)** ^∗^	0 (0.00)	0 (0.00)	1 (9.09)	3 (5.26)	1 (1.75)	4 (5.00)	3 (3.80)
Brain stem (%)	0 (0.00)	0 (0.00)	0 (0.00)	0 (0.00)	0 (0.00)	2 (3.51)	0 (0.00)	1 (1.27)
PIVH (%)	0 (0.00)	0 (0.00)	0 (0.00)	**1 (9.09)** ^∗^	0 (0.00)	0 (0.00)	0 (0.00)	1 (1.27)
Right-sided hematoma (%)	24 (46.15)	23 (47.92)	17 (44.74)	2 (18.18)	27 (47.37)	27 (47.37)	33 (41.25)	36 (45.57)
Insular involvement (%)	8 (15.38)	**14 (29.17)** ^∗^	2 (5.26)	0 (0.00)	**10 (17.54)** ^∗^	**13 (22.81)** ^∗^	9 (11.25)	6 (7.59)
SIVH (%)	**27 (51.92)** ^∗^	**20 (41.67)** ^∗^	14 (36.84)	2 (18.18)	16 (28.07)	18 (31.58)	**30 (37.50)** ^∗^	22 (27.85)
Hematoma volume > 30 ml (%)	**35 (67.31)** ^∗^	**28 (58.33)** ^∗^	12 (31.58)	**11 (90.91)** ^∗^	**31 (54.38)** ^∗^	30 (52.63)	**43 (53.75)** ^∗^	36 (45.57)
Leukocytosis (%)	3 (5.77)	2 (4.17)	0 (0.00)	0 (0.00)	0 (0.00)	3 (5.26)	3 (3.75)	**0 (0.00)** ^∗^
Low platelet count (%)	0 (0.00)	1 (2.08)	1 (2.63)	0 (0.00)	1 (1.75)	1 (1.75)	1 (1.25)	0 (0.00)
Abnormal hemoglobin level (%)	1 (1.92)	1 (2.08)	0 (0.00)	**2 (18.18)** ^∗^	1 (1.75)	1 (1.75)	2 (2.50)	1 (1.28)
Coagulation abnormalities (%)	0 (0.00)	0 (0.00)	0 (0.00)	0 (0.00)	0 (0.00)	0 (0.00)	0 (0.00)	1 (1.28)
Mortality (%)	3 (5.77)	5 (10.42)	3 (7.89)	4 (36.36)	6 (10.53)	8 (14.04)	8 (10.00)	10 (12.66)

ECG: electrocardiogram; ICH: intracerebral hemorrhage; SD: standard deviation; Time: time of onset on admission; GCS: Glasgow Coma Scale; PIVH: primary intraventricular hemorrhage; SIVH: secondary intraventricular hemorrhage; QTc: corrected QT interval; LBBB: left bundle branch block; CK: creatine kinase; CKMB: creatine kinase-myocardial subfraction; LDH: lactic dehydrogenase; hs-cTnT: high-sensitive cardiac troponin T.

**Table 3:** Univariate analysis of significant ECG changes in ICH. Significant values (*p* < 0.05) are in bold.

Variable	Entire cohort (*n* = 208)	QTc prolongation (*n* = 52)	*p* value	ST depression (*n* = 48)	*p* value	Inverted T wave (*n* = 38)	*p* value	LBBB (*n* = 11)	*p* value
Age (SD)	61.23 (13.64)	60.37 (13.49)	0.601	61.79 (12.38)	0.576	66.92 (13.50)	**0.006** ^∗^	60.18 (11.80)	0.77
Male	136 (65.38)	34 (65.38)	1.000	33 (68.75)	0.808	20 (52.63)	0.068	8 (72.73)	0.876
History of hypertension	140 (67.31)	37 (71.15)	0.495	33 (68.75)	0.849	28 (73.68)	0.127	9 (81.82)	0.644
History of type 2 diabetes	16 (7.69)	6 (11.54)	0.229	4 (8.33)	0.233	2 (5.26)	0.776	1 (9.09)	1
History of cerebrovascular diseases	32 (15.38)	9 (17.31)	0.657	10 (20.83)	0.692	11 (28.95)	**0.01** ^∗^	1 (9.09)	0.858
History of coronary heart disease	11 (5.29)	2 (3.85)	0.592	2 (4.17)	0.617	4 (10.53)	0.232	2 (18.18)	0.208
History of smoking	76 (36.54)	19 (36.54)	1.000	19 (39.58)	0.806	11 (28.95)	0.282	3 (27.27)	0.72
History of drinking	45 (21.63)	11 (21.15)	0.923	11 (22.92)	0.576	4 (10.53)	0.066	2 (18.18)	1
History of antiplatelet drugs	14 (6.73)	5 (9.62)	0.338	5 (10.42)	0.245	7 (18.42)	**0.001** ^∗^	0 (0.00)	0.360
History of anticoagulant drugs	1 (0.48)	0 (0.00)	0.563	1 (2.08)	0.067	1 (2.63)	0.410	0 (0.00)	0.813
Time (IQR)	6 (21)	5.00 (18.25)	0.205	6.50 (17.50)	0.810	6 (21)	0.255	5 (5)	0.2
GCS ≤ 8	60 (28.85)	32 (61.54)	0.077	27 (56.25)	**0.009** ^∗^	24 (63.16)	0.229	3 (27.27)	**0.003** ^∗^
Blood glucose (IQR)	7.43 (4.04)	7.93 (4.01)	0.129	7.94 (5.43)	0.061	7.065 (3.60)	0.902	10.39 (5.34)	0.104
Basal ganglia	100 (48.08)	26 (50.00)	0.749	21 (43.75)	0.494	8 (21.05)	**<0.001** ^∗^	8 (72.73)	0.099
Lobar	62 (29.81)	18 (34.62)	0.381	16 (33.33)	0.543	2 (5.26)	**<0.001** ^∗^	1 (9.09)	0.221
Thalamus	28 (13.46)	7 (13.46)	1.000	11 (22.92)	**0.029** ^∗^	28 (73.68)	**<0.001** ^∗^	0 (0.00)	0.368
Cerebellum	11 (5.29)	0 (0.00)	**0.049** ^∗^	0 (0.00)	0.062	0 (0.00)	0.226	1 (9.09)	1
Brain stem	4 (1.92)	0 (0.00)	0.244	0 (0.00)	0.269	0 (0.00)	0.763	0 (0.00)	1
PIVH	1 (0.48)	0 (0.00)	0.563	0 (0.00)	0.583	0 (0.00)	1	1 (9.09)	**0.046** ^∗^
Right-sided hematoma	96 (46.15)	24 (46.15)	1.000	23 (47.92)	0.780	17 (44.74)	0.846	2 (18.18)	0.052
Insular involvement	20 (9.62)	8 (15.38)	0.103	14 (29.17)	**<0.001** ^∗^	2 (5.26)	0.482	0 (0.00)	0.552
SIVH	56 (26.92)	27 (51.92)	**<0.001** ^∗^	20 (41.67)	**0.009** ^∗^	14 (36.84)	0.127	2 (18.18)	0.733
Hematoma volume > 30 ml	87 (41.83)	35 (67.31)	**<0.001** ^∗^	28 (58.33)	**0.008** ^∗^	12 (31.58)	0.157	11 (90.91)	**0.001** ^∗^
Leukocytosis	8 (3.85)	3 (5.77)	0.405	2 (4.17)	0.895	0 (0.00)	0.173	0 (0.00)	0.495
Low platelet count	2 (0.96)	0 (0.00)	0.412	1 (2.08)	0.364	1 (2.63)	0.243	0 (0.00)	0.737
Abnormal hemoglobin level	3 (1.44)	1 (1.92)	0.737	1 (2.08)	0.671	0 (0.00)	0.409	2 (18.18)	**<0.001** ^∗^
Coagulation abnormalities	2 (0.96)	0 (0.00)	0.412	0 (0.00)	0.436	0 (0.00)	0.502	0 (0.00)	0.737
Mortality	17 (8.17)	3 (5.77)	0.465	5 (10.42)	0.518	3 (7.89)	0.945	4 (36.36)	0.052

ECG: electrocardiogram; ICH: intracerebral hemorrhage; SD: standard deviation; Time: time of onset on admission; GCS: Glasgow Coma Scale; PIVH: primary intraventricular hemorrhage; SIVH: secondary intraventricular hemorrhage; QTc: corrected QT interval; LBBB: left bundle branch block.

**Table 4:** Univariate analysis of elevated myocardial enzymes in ICH. Significant values (*p* < 0.05) are in bold.

Variable	Entire cohort (*n* = 208)	Elevated CK (*n* = 57)	*p* value	Elevated CKMB (*n* = 57)	*p* value	Elevated LDH (*n* = 80)	*p* value	Elevated hs-cTnT (*n* = 79)	*p* value
Age (SD)	61.23 (13.64)	60.58 (13.63)	0.675	58.42 (13.57)	0.070	59.66 (14.20)	0.200	64.91 (14.81)	**0.003** ^∗^
Male (%)	136 (65.38)	42 (73.68)	0.122	43 (75.44)	0.061	49 (61.25)	0.322	51 (64.56)	0.844
History of hypertension	140 (67.31)	39 (68.42)	0.833	39 (68.42)	0.833	54 (67.50)	0.963	57 (72.15)	0.244
History of type 2 diabetes	16 (7.69)	5 (8.77)	0.720	4 (7.02)	0.822	4 (5.00)	0.249	11 (13.92)	**0.008** ^∗^
History of cerebrovascular diseases	32 (15.38)	12 (21.05)	0.164	10 (17.54)	0.596	10 (12.50)	0.362	14 (17.72)	0.465
History of coronary heart disease	11 (5.29)	3 (5.26)	0.992	4 (7.02)	0.494	5 (6.25)	0.624	7 (8.86)	0.072
History of smoking	76 (36.54)	22 (38.60)	0.705	25 (43.86)	0.178	28 (35.00)	0.716	28 (35.44)	0.797
History of drinking	45 (21.63)	10 (17.54)	0.379	13 (22.81)	0.801	15 (18.75)	0.424	16 (20.25)	0.705
History of antiplatelet drugs	14 (6.73)	7 (12.28)	0.050	7 (12.28)	0.050	7 (8.75)	0.358	10 (12.82)	**0.007** ^∗^
History of anticoagulant drugs	1 (0.48)	0 (0.00)	0.538	0 (0.00)	0.538	0 (0.00)	0.428	0 (0.00)	0.437
Time (IQR)	6 (21)	7 (21)	0.287	5 (6)	**0.039** ^∗^	5 (11)	0.167	6 (21)	0.452
GCS ≤ 8	60 (28.85)	37 (64.91)	0.222	30 (52.63)	**<0.001** ^∗^	49 (61.25)	**0.013** ^∗^	51 (64.56)	0.100
Blood glucose (IQR)	7.43 (4.04)	7.91 (3.43)	0.346	8.03 (4.09)	**0.02** ^∗^	7.97 (4.55)	**0.008** ^∗^	7.91 (4.61)	**0.024** ^∗^
Basal ganglia	100 (48.08)	27 (47.37)	0.900	26 (45.61)	0.662	42 (52.50)	0.313	31 (39.24)	**0.046** ^∗^
Lobar	62 (29.81)	20 (35.09)	0.306	18 (31.58)	0.732	23 (28.75)	0.792	28 (35.44)	0.164
Thalamus	28 (13.46)	6 (10.53)	0.446	10 (17.54)	0.289	11 (13.75)	0.923	14 (17.72)	0.159
Cerebellum	11 (5.29)	3 (5.26)	0.992	1 (1.75)	0.162	4 (5.00)	0.883	3 (3.80)	0.452
Brain stem	4 (1.92)	0 (0.00)	0.215	2 (3.51)	0.306	0 (0.00)	0.110	1 (1.27)	0.589
PIVH	1 (0.48)	0 (0.00)	0.538	0 (0.00)	0.538	0 (0.00)	0.428	1 (1.27)	0.200
Right-sided hematoma	96 (46.15)	27 (47.37)	0.829	27 (47.37)	0.829	33 (41.25)	0.262	36 (45.57)	0.895
Insular involvement	20 (9.62)	10 (17.54)	**0.017** ^∗^	13 (22.81)	**<0.001** ^∗^	9 (11.25)	0.527	6 (7.59)	0.439
SIVH	56 (26.92)	16 (28.07)	0.819	18 (31.58)	0.352	30 (37.50)	**0.007** ^∗^	22 (27.85)	0.814
Hematoma volume > 30 ml	87 (41.83)	31 (54.38)	**0.024** ^∗^	30 (52.63)	0.052	43 (53.75)	**0.006** ^∗^	36 (45.57)	0.392
Leukocytosis	8 (3.85)	0 (0.00)	0.076	3 (5.26)	0.514	3 (3.75)	0.955	0 (0.00)	**0.025** ^∗^
Low platelet count	2 (0.96)	1 (1.75)	0.472	1 (1.75)	0.472	1 (1.25)	0.736	0 (0.00)	0.271
Abnormal hemoglobin level	3 (1.44)	1 (1.75)	0.817	1 (1.75)	0.817	2 (2.50)	0.312	1 (1.28)	0.881
Coagulation abnormalities	2 (0.96)	0 (0.00)	0.383	0 (0.00)	0.383	0 (0.00)	0.261	1 (1.28)	0.714
Mortality	17 (8.17)	6 (10.53)	0.447	8 (14.03)	0.058	8 (10.00)	0.447	10 (12.65)	0.058

ICH: intracerebral hemorrhage; SD: standard deviation; Time: time of onset on admission; GCS: Glasgow Coma Scale; PIVH: primary intraventricular hemorrhage; SIVH: secondary intraventricular hemorrhage; CK: creatine kinase; CKMB: creatine kinase-myocardial subfraction; LDH: lactic dehydrogenase; hs-cTnT: high-sensitive cardiac troponin T.

## Data Availability

Data are available to researchers on request for purposes of reproducing the results or replicating the procedure by directly contacting the corresponding author.
